# Young People’s Understanding of Coercive Control in Northern Ireland

**DOI:** 10.1007/s40653-022-00508-8

**Published:** 2023-01-12

**Authors:** Susan Lagdon, Lucia Klencakova, Dirk Schubotz, Ciaran Shannon, Mark A. Tully, Cherie Armour, Julie-Ann Jordan

**Affiliations:** 1 School of Psychology, Ulster University, Northern Ireland, UK; 2 School of Social Sciences, Education and Social Work, Queen’s University, Belfast, UK; 3 IMPACT Research Centre, Northern Health and Social Care Trust, Northern Ireland, UK; 4 School of Health Sciences, Ulster University, Northern Ireland, UK; 5 School of Psychology, Queen’s University Belfast, Northern Ireland, UK

**Keywords:** Coercive Control, Intimate Partner Violence, Young People

## Abstract

Coercive control and related research have progressed significantly in the past number of years, with an ever-growing evidence base adding to its construct. However, currently there is a lack of evidence on young people’s knowledge and understanding of coercive control. We included a module of questions in the 2020 Northern Ireland Young Life and Life and Times survey (*n* = 2,069) with the aim of capturing baseline measurable data on understanding of coercive control within intimate relationships among 16-year olds. Only 16% (*n* = 325) of respondents had heard of the term coercive control and knew what it meant. Findings also revealed that females, compared to males, were less likely to have heard of coercive control. When the victim being subjected to the behaviours was portrayed as female as opposed to male there was stronger recognition of the associated risks, need for support, and the seriousness of the situation. Our study findings call to question young people’s knowledge of unhealthy intimate relationship behaviours beyond blatant and deliberate acts of harm such as those described in the coercive control scenarios. Gender disparities in awareness of coercive control across the study sample also give cause for concern given the increased risk of intimate partner violence among women and girls as well as lower reporting and help seeking among male victims. Results solidify the necessity for dedicated preventative and intervention efforts which focus on intimate relationships and reflect the diverse needs and experiences of young people. Supporting young people to act on their own behalf is an important step change to empowerment within their own intimate relationships.

## Introduction

Intimate partner violence (IPV) is a term used to describe violent and abusive behaviour perpetrated by a current or former partner. The term itself is often used synonymously with ‘domestic violence or abuse’, but the former offers a clear focus on the relationship dynamic between the perpetrator and the victim. Either term encompasses a number of tactics or acts which perpetrators may adopt in order to impose harm on another. These tactics or acts can be physical, sexual or psychological and emotional in nature (Patafio et al., 2022). The experience of IPV is seldom the result of a one off incident, with many victims subjected to multiple forms of violence within their relationship over a period of time. Indeed, newer terminology such as ‘coercive control’ is being more readily adopted to better describe the pattern and intent of such harmful behaviour perpetuated through psychological and emotional abuse (Lagdon et al., 2022). While the term ‘coercive control’ is considerate of all forms of violence, its intention is to better encapsulate the non-physical forms of abuse ‘which aim to intimidate, threaten and humiliate a person or restrict a person’s liberty’ through isolation, surveillance and micro regulation’ (Johnson, 2006; Lagdon et al., 2022; Policastro & Finn, 2021; Soliman, 2019).

Experiences of IPV or abuse are not limited to adult relationships. Increasing evidence has demonstrated that a significant proportion of young people can, and do, experience harm within their own intimate relationships (Barter et al., 2017). Research suggests that first occurrences of IPV can happen before the age of 15 and gradually increase in intensity and severity (see for instance Foshee et al., 2009; Gadd et al., 2015; Kennedy et al., 2018). It is estimated that 1 in 4 adolescent girls between the ages of 15 and 19 years of age have already been subjected to physical and/or sexual violence in their lifetime (WHO, 2021); less is known about adolescent males’ experiences in this regard. Reported IPV rates among young people in the United States (US) are between 20 – 29.4% (Foshee et al, 2013; Gehring & Vaske, 2017), 59.7% in Sweden (Korkmaz et al., 2020) and 66–75% within the United Kingdom (UK) (Barter et al., 2015). Differences in methodologies, target samples and measurement tools result in limited potential for country comparisons as well as accuracy in the rate of young people’s experiences of IPV. Furthermore, the prevalence of IPV may also vary within countries based on population density (Strand & Storey, 2019).

Previous research highlights that young people are exposed to different types of violence similar to adult relationships, as well as digital and technology-enabled abuse (Dank et al., 2014; Korkmaz et al., 2020; Patton et al., 2014). To date, the most prevalent categorisation of IPV within the research literature is still physical and sexual violence (Duval et al., 2018; Postmus et al., 2020); however, as previously noted, IPV can take many forms including non-physical forms of abuse such as those categorised under coercive and controlling behaviours. In a five-country European survey, emotional abuse was reported by approximately 50% of 3,277 young people who stated that they had been subjected to some form of emotional extortion and/or coercive behaviour from an intimate partner (Barter, 2018; Stonard et al., 2014). In the UK specifically, emotional abuse, sabotage, surveillance and abusers’ continued scrutiny or criticism was experienced by 30 – 50% of young men and 60 – 75% of young women (Barter, 2018; Wood et al., 2011). Similarly, Young and colleagues (2017) surveyed 1,751 students aged 16 –19 across six further education settings in England and Wales. The researchers reported that both male and females within the sample had experienced threating and controlling behaviour within a dating relationship (ibid).

According to Stark (2012), “The major outcome of coercive control is a hostage-like condition of entrapment that arises from the suppression of a victim’s autonomy, rights and liberties” (p. 5). The impact associated with these types of experiences include a decline in mental and physical health as well as a range of psychosocial challenges such as substance abuse, delinquency and aggression (see for instance Barter & Stanley, 2016; Dank et al., 2014; Foshee et al., 2013; Wiklund et al., 2010). Moreover, coercive and controlling actions and behaviours such as forced pregnancy, school sabotage and/or economic abuse can lead to a series of long-term, negative outcomes such as economic disadvantage, academic underachievement and even a cycle of abusive relationships (Banyard et al., 2017; Postmus et al., 2020; Voth Schrag & Edmonton, 2017). Cyber abuse, specifically, has been linked to serious drug use and aggression, truancy, disengagement and poor performance at school (Dank et al., 2014).

Coercive control and related research have progressed significantly in the past number of years, with an ever-growing evidence base adding to its construct. This is reflected in changing policy and legislation such as the addition of coercive control to the definitions of domestic violence and abuse within the UK and Ireland. This expansion has led to targeted approaches to prevention and intervention including ‘domestic violence’ awareness raising among school-aged individuals (Stanley et al., 2015) although limits to success have been cited. This is perhaps due to the interventions being based on research evidence from adult samples, limited co-production with young people underpinning design, catch-all programmes with too broad a scope (addressing domestic abuse including family violence), gender focused descriptions of IPV scenarios (and therefore perceived as exclusionary) (Fox et al., 2014) as well as language barriers and limited relevancy to the intended target population (Korkmaz, 2018).

Indeed, the field of IPV research is saturated with terminology to describe this experience reflective of time, context and culture in which it has occurred. Hickman et al. (2004) notes that terms such as ‘domestic abuse’ often relate to married or cohabitating adult couples and are therefore not relatable to young people and their relationship dynamics; hence, we must adopt youth-specific concepts (Korkmaz, 2018). These disparities in terminology risk engagement of young people in IPV programmes, as they do not recognise themselves in ‘adult’ situations. This risk is further exacerbated when we add covert forms of abuse such as coercive control. This form of abuse is also known as the ‘hidden’ form of IPV (Stark, 2012) which can often be misunderstood as a sign of care, ultimately preventing young people from perceiving themselves as victims and therefore hindering help seeking behaviours, including reporting to the authorities (Abbott et al., 2021; Hellevik et al., 2015). Currently there is a lack of evidence on young people’s knowledge and understanding of coercive control. Without clear understanding of coercive control in the context of young adult relationships, young adults remain at risk and support and best guidance in this regard is difficult to develop.

### Study Aims

To address the need for evidence-based knowledge to improve young people’s awareness and victim responding to coercive control, a module of questions was included in the 2020 Young Life and Life and Times (YLT) survey with the aim of capturing baseline measurable data on understanding of coercive control within intimate relationships. The study also explored the impact of victim gender on young people’s attitudes towards coercive control behaviours within intimate relationships, and aimed to identify predictors of coercive awareness amongst young people. Covariates were chosen based on their potential contribution towards IPV attitudes and risk of IPV as highlighted by Yang et al. (2021) as well as insights from Strand et al. (2019).

## Methods

The YLT survey is a cross-sectional survey undertaken annually since 2003. Fieldwork for the 2020/21 YLT survey took place in May 2021. The survey sample was drawn from the UK’s Child Benefit Register which is held at Her Majesty’s Revenue and Customs (HMRC). A statutory instrument is in place which allows the Access Research Knowledge (ARK) social policy hub to access the Child Benefit Register for its annual YLT survey. Child Benefit is a welfare benefit paid in the UK to parents or caregivers bringing up children and this is paid for each child. Whilst means testing for Child Benefit payments was introduced by government in April 2013, because this is administered via taxation of higher earners, the Child Benefit Register remains de facto a universal sample frame for 16-year olds and it is therefore ideal for the YLT survey. A sample of 5,000 randomly selected young people in Northern Ireland who celebrated their 16th birthday in April, May or June was selected for the 2020/21 survey and was provided to ARK directly by HMRC via secure data transfer following the agreement of a Service Level Agreement (SLA) and Memorandum of Understanding (MoU) which was signed between ARK and HMRC.

Data for the current study was collected via an online survey. The programme for the survey was written by the Centre for Data Digitisation and Analysis (CDDA) at Queen’s University Belfast, and the survey tool and data were held securely on the ARK server at Ulster University. Postal completion was offered as an alternative mode of taking part in the survey. Three 16-year olds requested a paper version of the survey, but none returned a completed paper questionnaire.

Each eligible respondent received an information letter which included the link to the online survey, a unique ID number, which was required to access the survey, as well as information on how to opt out of taking part. Reminder letters were sent out after 10 days to every respondent who had not opted out but had also not completed the survey. A mobile phone helpline was maintained throughout the fieldwork period. All respondents were offered a £10 shopping voucher for completing the YLT survey, and these were sent out after the fieldwork was completed.

After opting out and removing young people from the sample who could not be contacted because their postal addresses were incorrect 4,913 eligible names and addresses remained. 2,147 young people logged onto the survey platform with their ID. ID numbers were disabled once a respondent had reached the end of the survey, ensuring that respondents could not complete the survey multiple times or pass on their ID number to someone else. After removal of the most incomplete responses (i.e. responses where only very few or no questions were completed), 2,069 responses remained. This represents an overall response rate of 42.2%.

### Measures

#### Demographic Questions

##### Gender identity

Participants’ responses to the gender were categorised as male, female and other (e.g. male to female transgender; female to male transgender).

##### Urban/rural

Survey respondents were asked to select which of five categories best described where they lived. Responses were then categorised as either urban (a big city; the suburbs or outskirts of a big city; a small city or town), rural (a country village; a farm or home in the country) or don’t know.

##### Ethnic minority

Assessed via the question ‘do you consider yourself to a member of a minority ethnic community’ (yes/no).

##### Financial status

Four categories were used to represent how well-off financially participants considered their families to be: 1) not at all well off/not very well off; 2) average; 3) well-off/very well off; 4) don’t know.

#### Coercive Control Questions

The YLT survey is run in a modular format, which means that the survey contains a range of questions and topics each year. Questions on coercive control were asked for the first time in YLT 2020/21. The development of these questions was informed by the findings of a consultation with a range of stakeholders with responsibility for relevant policy and service provision in Northern Ireland. These were from both the statutory sector (such as Northern Health and Social Care Trust; Adult Mental Health and Children services; and Department of Justice Northern Ireland), and non-governmental organisation sector (including Causeway Women’s Aid; Barnardo’s Northern Ireland; and Nexus Northern Ireland). YLT respondents were presented with a relationship scenario focusing on coercive control within intimate heterosexual relationships (Full module details can be access from https://www.ark.ac.uk/ylt/2020_21/YLTquest2020_21A.pdf). Respondents were randomly allocated to one of two groups where the perpetrator was presented as male and the victim female (version 1) or perpetrator was female, and victim was male (version 2). Same-sex and gender-variant scenarios were also considered at design stage, but could not be accommodated due to space restrictions in the questionnaire.

Following each scenario, respondents indicated their level of agreement or disagreement using a 5-point scale (1 = strongly disagree, 2 = disagree, 3 = neither agree nor disagree, 4 = agree and 5 = strongly agree) to ten statements covering attitudes towards: coercive and controlling behaviours; victims of coercive control; talking about coercive control; and whether coercive control is a crime. Respondents were also asked if they had previously heard of the term ‘coercive control’, with response options ‘yes, and I know what it means’, ‘yes, but I am unsure what it means’ and ‘no’. For all questions in this module, respondents also had the option to say that they ‘don’t know’, or that they ‘prefer not to say’.

### Data Analysis

All data analyses were carried out using SPSS Statistics Version 26. The demographic composition of samples A (female victim) and B (male victim) were compared via chi-squared tests. A multinomial logistic regression model with ‘yes, and I know what it means’ as the reference category was used to examine predictors of coercive control awareness. Predictors incorporated in the models included gender identity (reference = female); urban/rural (reference = urban); ethnic minority (reference = no, not from an ethnic minority); and financial status (reference = Not at all well-off/not very well-off).

Multiple Analysis of Variance (MANOVA) assessed if agreement levels to each of the ten statements relating to attitudes towards coercive control varied by victim gender. A number of respondents replied ‘don’t know’ to the ten coercive control statements (1.3% – 13.9%) these responses were excluded from the statistical analysis, thereby allowing the data to be treated as continuous. Follow-up univariate ANOVAs where then used to determine which specific coercive control statements showed variation in agreement levels by victim gender. As missing data rates for the ten coercive control attitudinal statements (1.2–1.5%), coercive control awareness (1.8%) and the demographic variables (0.0 – 2.2%) were low, missing data was dealt with via listwise deletion.

### Ethics

Ethical approval for the 2020/21 YLT survey is in place from the Ethics Committee of the School of Social Sciences, Education and Social Work at Queen’s University Belfast where the YLT survey team is based.

## Results

### Sample Demographics

No significant differences were evident between the samples on the gender identification, urban/rural, ethnic minority, or financial status variables, suggesting samples A and B have comparable demographic profiles (Table 1).

**Table 1:** Demographic profiles of Samples A and B

Respondent characteristics		Sample A (female victim)		Sample B (male victim)		Sample A vs Sample B	
		n	%	n	%	x^2^	p
Gender identity	Male	452	44%	443	42%	2.76	.252
	Female	552	54%	596	57%		
	Other	16	2%	10	1%		
Urban/Rural	Urban	645	63%	676	64%	.65	.723
	Rural	351	34%	346	33%		
	Don’t know	23	2%	27	3%		
Ethnic minority	Yes	125	12%	113	11%	.92	.338
	No	878	88%	906	89%		
Financial status	Not at all well-off/not very well-off	116	11%	116	11%	.68	.877
	Average	489	48%	504	48%		
	Well-off/very well-off	337	33%	340	32%		
	Don’t know	76	7%	88	8%		

Only 16% (*n* = 325) of respondents had heard of the term coercive control and knew what it meant. The remainder either said they had heard of it but were unsure what it meant (*n* = 483; 24%) or had not heard of the term at all (*n* = 1223; 60%). A multinomial logistic regression revealed that females were more likely than males to say ‘yes, but I am unsure what it means’ or ‘no’, rather than stating that they had heard of coercive control and knew what it meant. In fact, 19% (*n* = 171) of males claimed to know what coercive control means, in contrast to 13% (*n* = 146) females. Similarly, females were more likely than those who self-reported their gender as ‘other’ to say they had never heard of the term coercive control as opposed to knowing to knowing what it meant (see Table 2).

**Table 2:** Multinomial logistic regression model using respondent characteristics to predict awareness of coercive control

Coercive Control Awareness category	Predictor	b	SE	Wald (df)	p	OR
Yes, but I am unsure what it means	Gender (male)	-0.72	0.15	23.20 (1)	< .001	0.49
	Gender (other)	-0.94	0.53	3.14 (1)	0.077	0.39
	UrbanRural (rural)	-0.10	0.15	0.40 (1)	0.527	0.91
	UrbanRural (don’t know)	0.99	0.66	2.20 (1)	0.138	2.68
	Ethnic minority (yes)	-0.20	0.25	0.63 (1)	0.427	0.82
	Financial Status (average)	0.11	0.23	0.23 (1)	0.629	1.12
	Financial Status (well/very well off)	0.20	0.25	0.67 (1)	0.414	1.22
	Financial Status (Don’t know)	0.02	0.38	0.00 (1)	0.953	1.02
No	Gender (male)	-0.39	0.13	9.47 (1)	0.002	0.67
	Gender (other)	-1.34	0.49	7.51 (1)	0.006	0.26
	UrbanRural (rural)	-0.10	0.13	0.55 (1)	0.457	0.91
	UrbanRural (don’t know)	1.01	0.62	2.70 (1)	0.101	2.75
	Ethnic minority (yes)	0.31	0.21	2.19 (1)	0.139	1.36
	Financial Status (average)	0.21	0.20	1.11 (1)	0.292	1.24
	Financial Status (well/very well-off)	0.21	0.21	0.94 (1)	0.332	1.23
	Financial Status (Don’t know)	0.68	0.32	4.54 (1)	0.033	1.97

Cox & Snell = .03; Nagelkerke = .03; McFadden = .01

### Attitudes Towards Coercive Control by Victim Gender

A MANOVA was used to compare agreement levels to the coercive control survey statements by victim gender. Using Wilks’ Lambda (Ʌ = 0.94, F (10, 1475) = 9.17, p < 0.001, partial eta squared = 0.06) a significant moderate sized effect of victim gender on attitudes towards the coercive control statements was evident. Subsequently, separate univariate ANOVAs were used to compare agreement levels by victim gender on each of the ten coercive control attitudinal variables. A Holm-Bonferroni correction (Holm, 1979) was applied to adjust for multiple comparisons.

When the victim was portrayed as female rather than male, there was significantly greater agreement that the victim would feel frightened; there was a future risk of physical harm; the victim should tell their friends and family about their partner’s behaviour; friends and family would consider the behaviour to be domestic abuse, the victim should report the behaviour to the police; the police would view the behaviour as criminal; and the behaviour should be viewed as a crime. Partial eta-squared for all effects ranged from 0.0–0.3, representing small effect sizes (see Table 3).

**Table 3:** ANOVAs comparing agreement ratings to survey statements by victim gender

	Female victim		Male victim					
	Mean	SD	Mean	SD	*F*	df	p	η_p_^2^
1. Commonplace	1.80	1.03	1.84	1.05	0.62	1, 1484	.430	.00
2. Frightened	4.62	0.60	4.43	0.72	24.98	1, 1484	< .001*	.02
3. Physical harm	4.30	0.71	4.01	0.83	40.09	1, 1484	< .001*	.03
4. Mental health	4.76	0.53	4.72	0.57	1.97	1, 1484	.160	.00
5. Tell friends & family	4.66	0.62	4.58	0.68	7.59	1, 1484	.006*	.01
6. Friends/family consider it domestic abuse	4.53	0.68	4.40	0.80	11.40	1, 1484	.001*	.01
7. Report to police	4.31	0.86	3.97	1.01	36.00	1, 1484	< .001*	.02
8. Police view behaviour as criminal	4.01	1.03	3.71	1.10	31.33	1, 1484	< .001*	.02
9. Behaviour is domestic abuse	4.36	0.79	4.28	0.83	5.04	1, 1484	.025	.00
10. Behaviour should be a crime	4.41	0.76	4.18	0.93	20.08	1, 1484	< .001*	.01

^*^Significant after Holm-Bonferroni correction applied to adjust for multiple comparisons

## Discussion

Research exploring the experience of IPV among young people is ever emerging, providing greater insight and understanding to abuse context and strategies among this group. What is agreed thus far is that the experience of IPV within early relationships can significantly increase risk to psychosocial development, and negatively impact mental health as well as subsequent intimate relationships. Preventive strategies targeted at young people and their intimate relationships to date have predominantly been delivered within a school-based setting under a relationship and sexual education agenda, although limited evidence is available on how such education addresses topics such as coercive control.

Current study findings have shown that only one in six (16%) 16-year olds in Northern Ireland report having heard of the term coercive control and having some understanding of its meaning. This is considerably lower than prevalence rate of coercive control awareness found in the adult population (64%; Lagdon et al., 2022). Encouragingly, many respondents did agree that the scenarios (whether with a male or female victim) were abusive and likely to result in negative outcomes. These findings are therefore twofold, young people taking part in the survey comprehend the abusive nature of the obvious and deliberate coercive and controlling behaviours described, but they do not recognise the terminology related to this and therefore the important nuances which separate a ‘normal’ relationship from coercive and controlling one. Indeed, for many young people there is a fine line between violence or control and ‘acts of passion or care’ (Barter, 2018; Harland & McCready, 2012). This highlights a significant gap in the links between knowledge and understanding of this form of abuse, (particularly identifying the early and more subtle signs), with significant potential to stifle future help seeking behaviour.

Additionally, findings also revealed that females, compared to males, were less likely to have heard of the term coercive control and know what it means (19% Vs 13%). Whilst we did not ask further questions regarding what is known about IPV, these findings remain a concern given that young women represent a higher proportion of IPV victims (Barter, 2009; Stermac et al., 2018). Interestingly, differences in sample agreement about the IPV experience and outcomes depending on victim gender were also observed. This included greater agreement about the impact on a female victim, their risk of future harm, if family and friends would believe them and if they should report to the police, compared to a male victim. Research suggests that only 17% of young victims choose to disclose their abuse experiences, particularly to an adult, with an even smaller number disclosing to formal sources including police services, which is especially true for young men (Bundock et al., 2018; Hellevik et al., 2015; Menna & Ruck, 2004). Young people are more likely to speak with peers about their experience (Hellevik et al., 2015) which is a quandary in the current context when we are unclear about if they know what coercive control is, or where to get support.

### Implications and Future Research

The lack of awareness of coercive control found in our sample of young people provides a strong rationale for building on work described by Stanley et al. (2015) to develop and evaluate early educational interventions which focus on all elements of both healthy and unhealthy relationships. “Knowing the signs of a healthy relationship is an important mediator towards identifying unhealthy and harmful behaviours, as is knowing and navigating support services if they are needed”. (Lagdon et al., 2021, p.4). Providing young people with the language and tools to communicate with parents, guardians, peers about unhealthy relationship practices will create wider pathways to support. Relatedly, bystander awareness with parents, guardians, peers and youth focused professionals will support a readiness to respond.

Other important preventative strategies are public awareness campaigns focusing on what coercive control means and signposting victims and their friends and family to appropriate courses of action and sources of support. Such campaigns have been reported as a strategy to prevent domestic violence (e.g. Gadomski et al., 2001), but to our knowledge this has never included coercive control, and there is also a lack of evidence regarding the effectiveness of these campaigns (Campbell & Manganello, 2006). The introduction of coercive control legislation in many parts of the world (including Northern Ireland where the survey was carried out) seems like a good opportunity to develop and evaluate such approaches. It would also seem important and helpful that all types of preventive strategies, including educational and public awareness campaigns, include young people in their design and delivery and provide information in a clear and accessible form. The increased usage of online social media as means for perpetration among young people is also an important consideration for such future awareness raising campaigns.

Given the prevalence of IPV within young people’s relationships coupled with the relative lack of awareness of coercive control, it is important that all professionals interacting with young people receive training on recognising and responding to all form of IPV including coercive control. These include police officers (Millar et al., 2021), teachers (Davies & Berger, 2019) and health care professionals (Turner et al., 2017). More broadly.

### Limitations

A number of limitations should be noted when interpreting the findings including consideration of context. The questions regarding coercive control were co-produced for the survey with stakeholders. How these generalise to other contexts would need further explored. In addition, the YLT is a cross-sectional representative sample. Longitudinal data is required to further examine the nature of the associations observed over time. Relatedly, a number of psychosocial factors may be associated with knowledge and understanding of IPV (including coercive control), future research should capture such information. Finally, greater diversity in sample characteristics and in study scenarios would also offer further insight into the perceptions of coercive and controlling behaviours beyond heterosexual relationships.

## Conclusion

For many of us, our first intimate relationship provides the opportunity and space to explore and learn what it means to be an intimate partner. Much of the literature focused on relationship success make reference to the importance of good communication, mutual respect and emotional readiness as a solid foundation of healthy relationship development (Stanley et al., 2020; Atkinson, 2005). The current study results call to question young people’s knowledge of unhealthy intimate relationship behaviours beyond blatant and deliberate acts of harm such as those described in the coercive control scenarios. Additionally, results indicate that higher number of females are maybe unaware of the term and meaning of coercive control and that young men are perceived as being at lower risk of harm. This is concerning given the increased risk of intimate partner violence among women and girls (WHO, 2021) as well as lower reporting and help seeking among male victims (Walker et al., 2020).

The development of legislation addressing coercive control is a welcome development across the UK and Europe but much of what we currently understand about violence and abuse has derived from the adult-focused literature. Presently, we are unable to gauge the extent of this issue among young people but what we do know is that coercive control is a feature in some young adults’ relationships (Barter, 2009). Researchers and policy makers continue to debate how to optimally define and respond to coercive control (Stark & Hester, 2019). The YLT survey results demonstrate that it is imperative that our dedicated preventative and intervention efforts reflect the diverse needs and experiences of young people. Supporting young people to act on their own behalf is an important step change to empowerment within their own intimate relationships.

## Data Availability

The survey data is freely available and be accessed from https://www.ark.ac.uk/ARK/ylt/datasets.
